# Design of a Compact Wireless Multi-Channel High Area-Efficient Stimulator with Arbitrary Channel Configuration

**DOI:** 10.3390/mi9010006

**Published:** 2017-12-27

**Authors:** Yuwei Zhang, Deng Luo, Ting Ou, Zhangyi Yuan, Heng Huang, Ling You, Yin Yue, Milin Zhang, Dongmei Li, Guolin Li, Kexin Yuan, Zhihua Wang

**Affiliations:** 1 Department of Electronic Engineering, Tsinghua University, Rohm Building, Beijing 10084, China; zhangyw15@mails.tsinghua.edu.cn (Y.Z.); huanghen16@mails.tsinghua.edu.cn (H.H.); 2 Institute of Microelectronics, Tsinghua University, Beijing 10084, China; luod14@mails.tsinghua.edu.cn (D.L.); ot15@mails.tsinghua.edu.cn (T.O.); zy-yuan16@mails.tsinghua.edu.cn (Z.Y.); guolinli@tsinghua.edu.cn (G.L.); zhihua@tsinghua.edu.cn (Z.W.); 3 Department of Biomedical Engineering, Tsinghua University, Beijing 100084, China; youl16@mails.tsinghua.edu.cn (L.Y.); yuey17@mails.tsinghua.edu.cn (Y.Y.); kexinyuan@tsinghua.edu.cn (K.Y.); 4 Research Institute of Tsinghua University in Shenzhen, Shenzhen 518057, China

**Keywords:** multi-channel neural stimulator, implantable medical device, functional electrical stimulation, arbitrary channel combination, programmable current generator

## Abstract

This paper presents the design of a wireless, implantable, multi-channel, programmable stimulator with arbitrary channel combination. A novel channel management module using a switch array is presented, enabling arbitrary channel configuration with a silicon area reduction of 81%. The chip was fabricated in a 0.18-μm Taiwan semiconductor manufacturing company (TSMC) high voltage (HV) complementary metal–oxide semiconductor (CMOS) technology. A stimulator system was realized using the proposed integrated circuit (IC). A wireless communication link was established between a specified Android-based graphical user interface (GUI) and the proposed device for control of the stimulation pattern and wireless battery charging. The size of the entire system occupies a volume of only 14 mm × 14 mm × 4 mm (without the battery). Experimental results demonstrated a successful independent configuration between different channels, as well as an arbitrary channel combination, as expected.

## 1. Introduction

**Figure 1:**
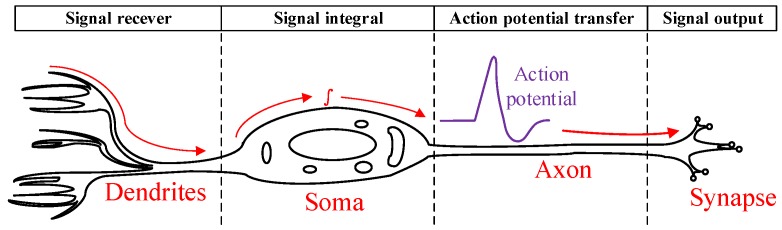
Sketch map of a single neuron, which consists of soma, dendrites, axon and synapse. The dendrites perform the function of signal reception, transmitting neural signals in the form of action potentials, while soma collects signals from all the dendrites.

The central nervous system is the seat of emotions, consciousness and thinking of human beings. It consists of neurons and neuroglia. Neuroglia deliver nutrition to neurons, while the neurons transfer bio-electricity to the whole body. As illustrated in Figure 1, a single neuron cell consists of: (1) soma, the main body of the neuron cell; (2) dendrites, the branched projections of the neuron cell; (3) axon, the long, slender projection of the neuron; and (4) the synapse. The neuron transfers the signal through dendrites, which act as signal receptors. A single neuron may have up to hundreds or thousands of dendrites. The collected signals are integrated in the soma, the center of the neuron. An action potential will be triggered to excite or suppress a target cell, when the signal reaches a threshold. Action potentials are transmitted along the axon. The longest axon may reach a few meters in length. The action potential will reach another neuron when it arrives at the synapse [1].

An implantable neural functional electrical stimulator (FES) is a device that generates artificial electrical pulses. The artificial electronic pulses will be transmitted to neurons to trigger or to suppress neural activities. FES has been used in various clinical practices, as well as frontier neuroscience research. The artificial electrical pulses can be applied to: (i) the brain, i.e., Deep Brain Stimulation (DBS) used for the treatment of Parkinson’s diseases, tremors and dystonia [2,3,4,5,6]; (ii) the central nervous system, i.e., Spinal Cord Stimulation (SCS) for pain suppression and for motor disorders [7,8,9,10]; (iii) the vision system, i.e., retinal prosthesis [11,12,13,14,15]; (iv) the trunk, i.e., stimulation of bladder for urinary disorder treatment [16,17,18,19].

The neuron acts as the load of a stimulator. An electrical model [1,20,21] is usually used for a quantitative analysis of the electrochemical energy transmission between the stimulator and the neuron. It models both the electrode and the tissue. At the electrode end, the charges accumulate at the interface between the electrode and the cell membrane, which acts as a capacitor, which isolates the electrode from the membrane during the stimulation. A leakage current always exists between the electrode and the membrane, with a value that varies with voltage. It can be modeled by a resistor parallel with the capacitor. The impedance of this model is time-variant, non-linear, anisotropic and inhomogeneous. Its value varies from a hundred Ohm to mega Ohm. Thus, a wide output voltage range is desired in current-mode stimulator designs.

In addition, safety is another significant consideration in stimulator design. There are two main safety concerns: the size of the device and charge accumulation. As an implantable device, the size is a key issue in clinical practice, especially for multiple channel applications, such as a retina prosthesis [22,23], or deep brain stimulation [6,24,25], Furthermore, the excess charge accumulation breaks the biochemical balance between cytoplasm and extracellular matrix, which will cause serious damage to the tissue. The nerve potential is evaluated by [26]:

(1) $$V_{X}=\frac{RT}{Z_{X}F}\ln\frac{C_{i,x}}{C_{o,x}}$$

where *X* stands for the specified ion type in the cell solution, such as potassium or sodium, *R* is the gas constant, which is equal to 8.314 (J/mol·K), *T* is the temperature in Kelvin, $Z_{X}$ is the valence of the ion, such as sodium, *F* is Faraday’s constant, which is equal to 9.6 × $10^{4}$ C/mol, and $C_{i,x}$ and $C_{o,x}$ stand for the intracellular and extracellular ionic concentration, respectively. According to this equation, the voltage increase due to charge residue has an effect on the concentration in the cell. It may cause unrecoverable distortion of the biochemical balance, which is denoted as electrochemical damage. Solutions to avoid charge residue have been proposed in the literature. The work in [27] proposed several approaches to solve the problem. There are two passive ways. The first one is by connecting all the electrodes to the ground or reference voltage after the stimulation pulse [28]. Another is inserting a large blocking capacitor in series with the electrode [29,30].

This paper proposes the design of a versatile, wireless, multi-channel, arbitrary multi-site, programmable stimulator. The programmable stimulator enables a high resolution stimulating pulse pattern with arbitrary channel configuration. A trade-off between the spatial resolution and silicon area consumption has been performed in the proposed work. A novel switch-based control method with eight independent pulse generators are integrated to realize an arbitrary site configuration. Capacitors are used to isolate the electrode from the pulse generator to reduce the charge residue. The system has a size of 14 mm × 14 mm × 3 mm without the battery.

The remainder of the paper is organized as follows. Section 2 presents the architecture of the multiple channel neural stimulation pattern generator design. Section 3 describes the architecture of the wireless multiple channel stimulator system. Section 4 illustrates the experimental results, while Section 5 concludes the entire work.

## 2. Architecture of the Multiple Channel Neural Stimulation Pattern Generator

**Figure 2:**
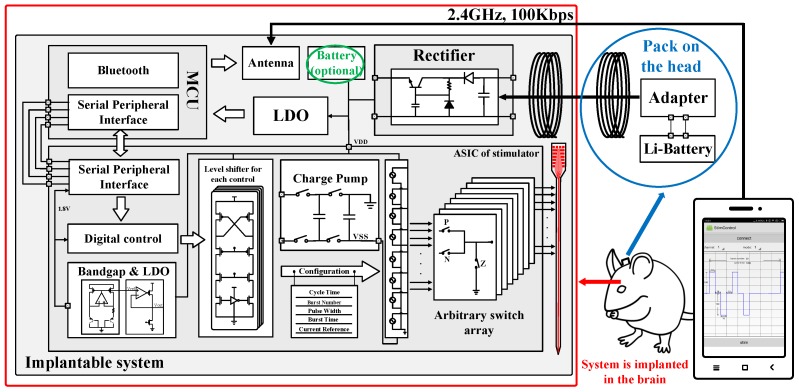
Block diagram of the overall system including the integrated proposed stimulator application-specific integrated circuit (ASIC) chip. The system consists of five parts: (i) the ASIC chip for multiple stimulation pattern generation, control and channel arrangement; (ii) an SPI-based interface connecting the external processing unit and the proposed ASIC; (iii) a Bluetooth-based wireless communication interface; and (iv) the wireless battery charging module. LDO: low dropout regulator.

The proposed application-specific integrated circuit (ASIC), as illustrated in Figure 2, consists of: (i) eight independent stimulation pairs; (ii) a switch array that used for arbitrary channel configuration; (iii) a charge pump for negative voltage generation; and (iv) digital circuits for the control of the pattern generation. An SPI module is also integrated in the digital circuits to serve as an interface to the external control unit.

### 2.1. Design of the Current Generator

**Figure 3:**
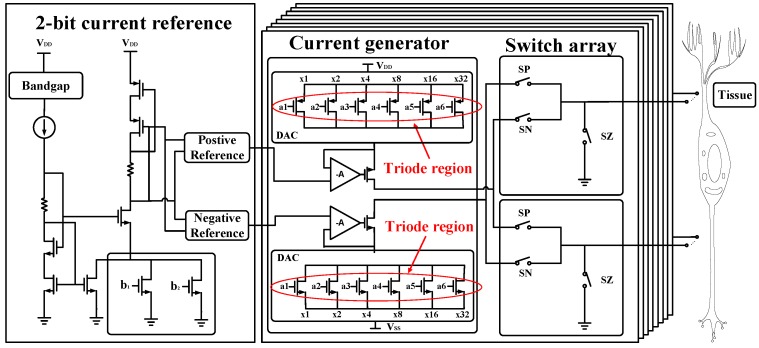
Architecture of the current generator used in the proposed stimulator. It consists of eight-pairs of current generators. Each generator integrates a 6-bit DAC for output current control. Two additional bits, $b_{1}$ and $b_{2}$, are used in the reference current mirror to vary the bias voltage. SP is the switch for positive phase, SN is the switch for negative phase, and SZ is the switch for zero phase.

A current generator is used to create constant current output for neural stimulation, as shown in Figure 3. Ideally, an infinite output impedance is achieved for a stable output. A current mirror is widely used for the current generating circuit, but suffers from a poor output impedance due to the channel length modulation. One common solution includes stacking transistors as cascode or using negative feedback, i.e., Wilson current mirror, at the expense of reduced voltage headroom. The works in [31,32] proposed a method to solve the trade-off between headroom and output impedance by biasing the transistor in the triode region as shown in Figure 3. The voltage over the resistor (of the transistors biased in the triode region) is stabilized by the amplifier, which defines the current. The output impedance are calculated as:

(2) $$R_{out}\;\left(Triode\right)=\left(A+1\right)g_{m}\;R_{on}\;r_{o}\;+\;r_{o}\;+\;R_{on}$$

where $g_{m}$ is the transconductance of the transistor, $R_{on}$ is the equivalent impedance of the triode region transistor and $r_{o}$ stands for the output impedance of the transistor. In addition, 6-bit DAC is integrated for the amplitude control of the output pulse waveform. The implementation consists of composing 6-bit weighting of triode transistor. All of the transistors are controlled by digital circuits through a level shifter. It is realized as a current digital-to-analog converter (current DAC) with an output current calculated as:

(3) $$I_{OUT}\;=I_{triode}\times \sum_{i=0}^{5}\left(2^{n}\times a_{n}\right)$$

where $I_{triode}$ is the stimulator output current and $a_{0}$ to $a_{5}$ are the bits of the current DAC. There are two additional bits, $b_{1}$ and $b_{2}$, used in the proposed design to control the reference current generated by the current mirror, which can vary depending on the application. Thus, a resolution of 8 bit is achieved in the stimulator current generator, enabling a current output varying from 8 μA to 2 mA. In addition, a voltage reference instead of current reference is used for its better precision.

### 2.2. Design of the Switch Array for Arbitrary Channel Combination

A switch array is included in to the proposed system to improve spatial resolution, as well as to enable an arbitrary combination between channels while minimizing area. Three extra switches are integrated with each stimulator.

**Figure 4:**
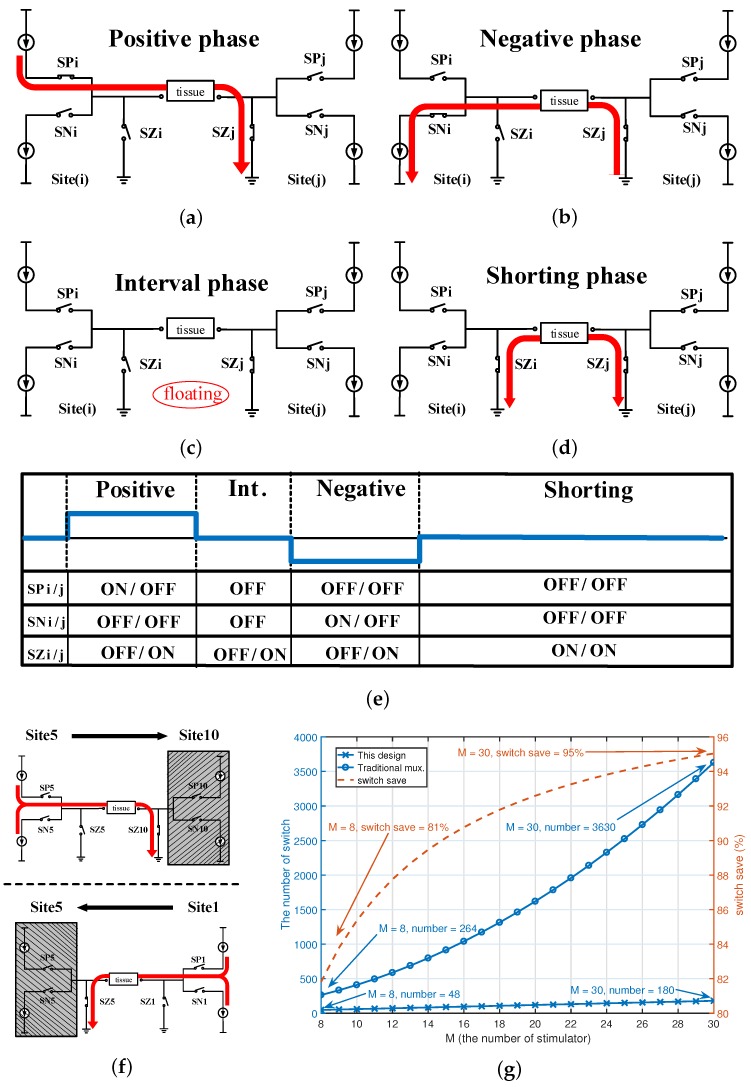
Architecture of the proposed switch array. There are three switches in each site, SP, SN and SZ. Four different phases, the (**a**) positive phase, (**b**) negative phase, (**c**) interval phase and (**d**) shorting phase can be realized by switching on/off the transistors in a pair of sites; (**e**) the configuration of the switches for different phases; (**f**) an example of the arbitrary switch array; (**g**) comparison between the total number of switches required in the traditional method (marked in circle) and the proposed one (marked with a cross), as well as the percentage of switches saved by the proposed method (dash line in red). Int. is the interval in Figure 4c.

The procedure begins with a configuration phase for the channel selection. As shown in Figure 4, for example, a pair of electrodes, site(i) and site(j), are selected from all 16 sites. Zero volts (GND) is used as a reference voltage. There are four states for a complete stimulating pulse generation: (1) positive phase, in which the current flows from site(i) to site(j); (2) negative phase, in which the current flows in the reverse direction from site(j) to site(i); (3) interval phase, in which no current is required; and (4) shorting phase, in which both sites are connected to the ground to release any charge left, as shown in Figure 4. Three switches, SP, SN and SZ, are needed for each site. A current path from site(i) to site(j) is realized in the positive state by turning on only SN${}_{i}$ and SZ${}_{j}$, while a reversal current path from site(j) to site(i) in the negative state by turning on only SP${}_{i}$ and SZ${}_{j}$. All switches are turned off in the interval state. Both of the SZ switches are turned on in the shorting state. An example is shown in Figure 4f. If a stimulating current is expected between site(5) and site(10). SP(10) and SN(10) are turned off with only SZ(10) turned on. Thus, site(10) is connected to the ground. Thus, the direction of the current between site(5), the tissue and site(10) can be controlled by turning on/off SP(5) and SN(5) in different phases.

Assume there are Mpairs of stimulator sites; the proposed scheme requires only 3 × 2 M switches in contrast to 2 × (2 M × M) + M switches if one would use a multiplexer. In this proposed design that consists of 16 channels used for eight stimulator sites, 81% of switches are saved, as compared to the traditional design with multiplexer switching. Figure 4f compares the total number of switches required in the traditional method and the proposed one. The percentage of switches saved by the proposed method increases when more sites are used.

### 2.3. Electrode Model Analysis

**Figure 5:**
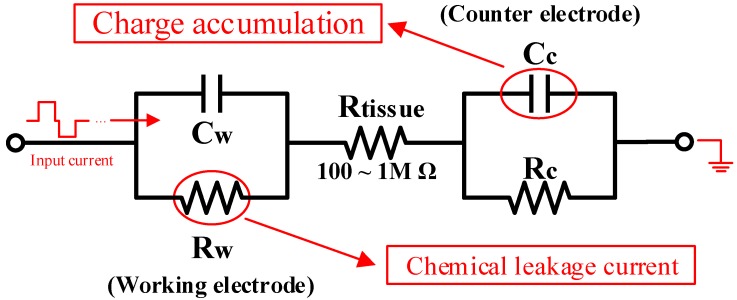
Electrode-electrolyte model. $C_{W}$, $R_{W}$ represent the equivalent impedance of the working electrode, which is denoted as the anode. $C_{C}$, $R_{C}$ represent the equivalent impedance of the counter electrode, denoted as the cathode.

According to the electrode model, as shown in Figure 5, $C_{W}$ and $C_{C}$ represent the charge accumulation on the electrode, electrolyte or cell membrane. $R_{W}$ and $R_{C}$ are the result of the leakage current caused by the electrochemical reaction. The subscripts W and C stand for the working side or counter side, respectively. Consider the fact that the stimulation frequency is usually lower than 1 kHz: $C_{C}$ can be consider as open circuit. Using the electrode model, one can calculate the voltage over the electrode-electrolyte model as follows:

(4) $$V=IR_{tissue}+\frac{\Delta tIR_{W}}{\Delta t\;+\;R_{W}C_{W}}$$

If tissue conductivity σ and the radius *a* are estimated, the impedance of the tissue can be calculated as:

(5) $$R_{tissue}=\frac{1}{4a\sigma }$$

and also the electrode $C_{W}$ can be calculated below with the electrode radius *a*:

(6) $$C_{W}=20\pi a{\left(cm\right)}^{2}\left(\mu F\right)$$

**Figure 6:**
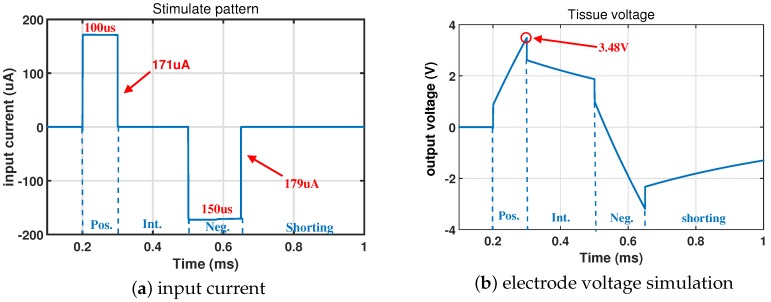
Simulation results of the electrode under the 6 nF working electrode capacitor $C_{W}$ and the 100 kΩ working electrode resistor $R_{W}$. (**a**) An input current with the 188 μA amplitude 011000 is applied; (**b**) the detected output voltage. Pos: positive, Neg: negative, Int. is the interval in Figure 4c.

Figure 6b shows the simulation result of the output voltage over the electrode model while a typical bipolar current stimulation, as shown in Figure 6a, is applied to the electrode-electrolyte model. One hundred kΩ of the $R_{W}$ working electrode resistance and 6 nF of the $C_{W}$ working electrode capacitor are used.

### 2.4. Design of the Charge Pump

**Figure 7:**
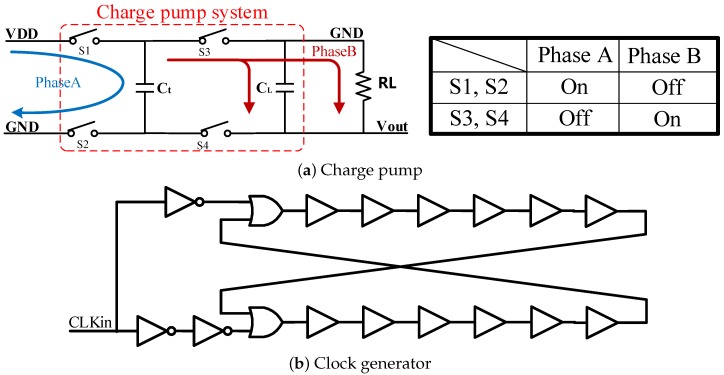
(**a**) Architecture of the charge pump design. There are two phases in the entire working flow: (i) Phase A is the input phase (highlighted in blue) while the battery charges the whole system; (ii) Phase B (highlighted in red) is the charge balancing between $C_{t}$ and $C_{L}$. (**b**) Architecture of the non-overlapping clock generator, which is used to meet the requirement for the loop control. VDD is the power voltage of the circuit and RL represents the load of the charge pump.

A two-phase negative voltage charge pump (CP) is designed, included in the proposed work, as illustrated in Figure 7a. It is used to generate the $V_{SS}$ of the stimulator. A fifty percent duty cycle, non-overlapping clock buffer is used. It pumps $V_{DD}$ to almost minus $V_{DD}$ with a driving capability of 1.5 mA.

Two functional phases are included, Phase A and Phase B. During Phase A, the battery charges capacitor $C_{t}$, while S1 and S2 are turned on. When the circuit is steady, the current pushed into $C_{t}$ can be calculated as:

(7) $$I_{A}=C_{t}\frac{V_{DD}-\left(0-{\bar{V}}_{out}\right)}{0.5t}$$

At Phase B, current flows out from $C_{t}$ as:

(8) $$I_{B}=C_{L}\frac{V_{ripple}}{0.5t}+\frac{\bar{V_{out}}}{R_{L}}$$

where ΔV is the voltage variation of $C_{L}$ during Phase A. It can be evaluated according to:

(9) $$C_{L}\frac{V_{ripple}}{0.5t}=\frac{\bar{V_{out}}}{R_{L}}$$

In Phase B, $C_{t}$ switches its role from the receiver to the power supply with the phase transform, which means the current flows out at Phase B all resources from the current charging at Phase A. Thus, the two currents are equal:

(10) $$I_{A}=I_{B}$$

According to Equations (7)–(10), $V_{out}$ can be calculated as:

(11) $$V_{out}=-V_{DD}\frac{1}{1\;+\;\frac{2}{R_{L}C_{t}f}}$$

where $R_{L}$ is the load impedance, $C_{t}$ is the transfer capacitor and *f* is the clock frequency. Since $\frac{1}{2R_{L}C_{t}f}$ is much smaller than two, the stable voltage is proportional to $R_{L}$, $C_{t}$ and *f*.

Another important aspect is the non-ideal rise time and fall time of the clock used for the switching timing control. The switches with a large size are required in the charge pump to pass a large amount of current. Thus, the rise time and the fall time will be large, due to the parasitic capacitance. The leakage path from $V_{DD}$ to the ground may be observed during the switching between the two phases. A non-overlapping clock generator is designed to avoid the leakage issue, as illustrated in Figure 7b.

### 2.5. Design of the Digital Control Logic

The full configuration of the stimulator requires several registers. Positive and negative pulse time ($T_{pos}$ and $T_{neg}$) and amplitude ($A_{pos}$ and $A_{neg}$) can be controlled independently. $T_{int}$ is the time between the positive and negative pulses. The time between two pulses is $T_{clr}\;+T_{int}$. Pulse numbers are determined by pulse group. The cycle time is $T_{clr}$. The details of the configuration are in Table 1.

## 3. Architecture of the Wireless Multiple Channel Stimulator System

The overall block diagram of the wireless multi-channel stimulator system is shown in Figure 2. In addition to the blocks described above, it also contains an SPI-based interface and a wireless power transfer module. These two blocks are described below.

### 3.1. Design of the Interface between the ASIC and Off-Chip Processing Unit

**Figure 8:**
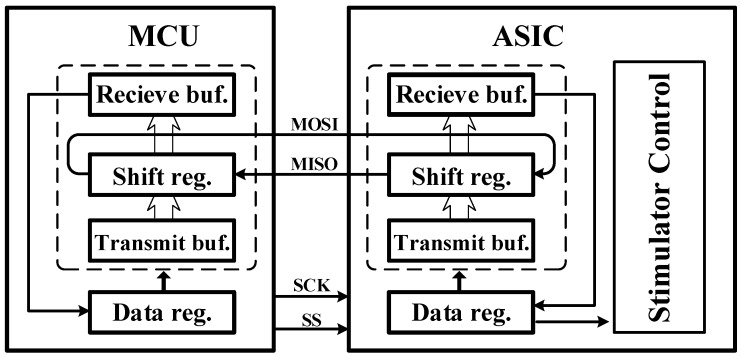
Block diagram of the SPI communication interface. The Master Output Slave Input (MOSI) and Master Input Slave Output (MISO) pins are used to receive and transmit data between the proposed ASIC and the off-chip devices. SCK, Serial Synchronous Clock. SS: slave select, buf.: buffer, reg.: regesiter.

A Serial Peripheral Interface (SPI) is a four-wire duplex, serial, synchronous communication protocol. As shown in Figure 8, the Master Output Slave Input (MOSI) and Master Input Slave Output (MISO) pins are used to receive and transmit data between the proposed ASIC and the off-chip devices. The SSN is a slave select pin output from the master and must be logical low during transmission. The SCK is the Serial Synchronous Clock used to control the timing and rate of data exchange. In addition, the Clock Polarity (CPOL) is a control bit to determine the SCK polarity; CPOL = 0 indicates that the SCK maintains as low during the idle state, otherwise SCK will be high. The Clock Phase (CPHA) is another control bit to decide the data sampling and data shifting at which clock edge. Sampling data is done at the odd edge when CPHA = 0, and shifting data are done at the even edge when CPHA = 0.

The main concept of SPI is exchanging data. The two shift registers in the master and slave module are linked to a circular shift register by the MOSI and MISO pins. Then, the circular shift register transmits 16-bit data bit by bit. The transmit buffer and receive buffer shown in Figure 8 are used to store the data, which are used to send or receive, respectively. In this design, an SPI slave module is used to transfer the stimulator control signals with a 16-bit shift register; the slave module is working in the most significant bit (MSB)-first mode, and CPOL and CPHA are set to zero.

### 3.2. Design of the Wireless Power Transfer Module

**Figure 9:**
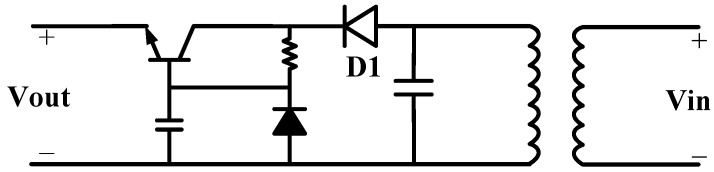
Architecture of the wireless power rectifier. A Schottky diode, $D_{1}$, is used.

An inductive coupled wireless power transmission is implemented in the proposed stimulator system. The receiver includes an inductively-coupled coil, a rectifier and a regulator. The transmitter is powered by a wireless adapter with oscillator and power amplifier. The power carrier frequency is 249 kHz. Figure 9 shows the wireless power receiver. A half-wave rectifier is implemented. The Schottky diode features a very low forward voltage drop, and its switching time is very short. When the transmitter is operating, the receiver coil produces an alternating current (AC) voltage. During the positive cycle, the diode is forward biased, and the voltage is passed through. During the negative half cycle, the diode is reversely biased, and the voltage will be zero. Therefore, the rectifier converts an AC voltage to a large ripple direct current (DC) voltage with low stability. A regulator, consisting of an NPN transistor and a Zener diode is used to provide a stable 5-V DC output.

## 4. Experimental Results

**Figure 10:**
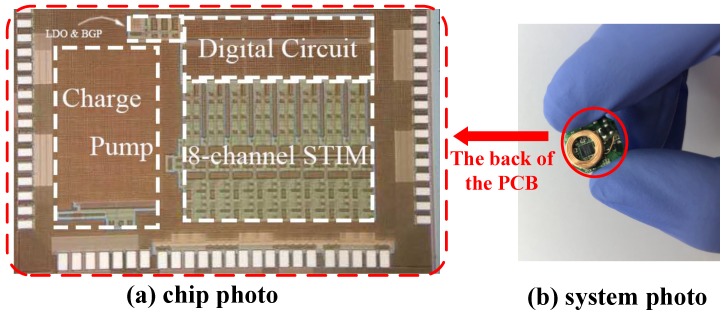
(**a**) Microphotograph of the proposed IC. The silicon area of the proposed chip is 1.5 mm× 2.5 mm. (**b**) Photograph of the proposed prototype system implemented using the described ASIC chip. The size of the proposed system is 14 mm × 14 mm × 3 mm excluding the battery.

The proposed ASIC has been fabricated in the Taiwan semiconductor manufacturing company (TSMC). 180 nm HV CMOS process, occupying a silicon area of 1.5 mm × 2.5 mm. Figure 10a shows the micrograph of the proposed chip. For each channel, the current generator and the charge pump occupy a silicon area of 140 μm × 848 μm and 1082 μm × 628 μm, respectively. A 500 pF capacitor, occupying 2 fF/μm${}^{2}$ in area, is included in the charge pump. A complete system was developed using the proposed ASIC for use as a wireless, compact 16-channel neural stimulator. The system has a radius of only 7 mm, as shown in Figure 10b. As described earlier, the system enables eight-channel programmable stimulation with arbitrary channel selection, wireless configuration and wireless charging.

**Figure 11:**
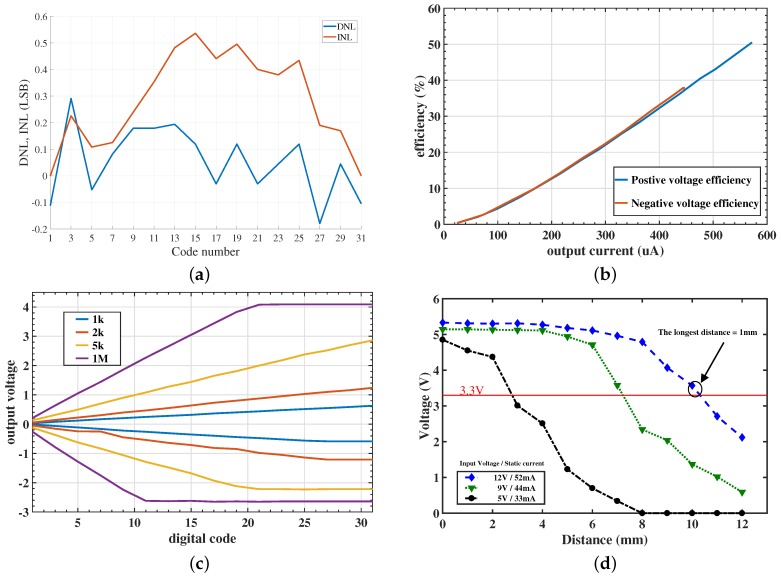
(**a**) Measured Differential Non-Linearity (DNL) and Integral Non-Linearity (INL) of the current DAC. The highest integral non-linearity of less than 0.6 LSB and differential non-linearity of less than 0.3 LSB are achieved. (**b**) The efficiency of a single channel stimulator. The result obtained under the load of 5 kΩ for different output currents. (**c**) The output current versus digital code. The positive voltage will saturate to the power rail, and the negative voltage saturation level will be determined by the charge pump. (**d**) The output voltage of the receiver end for different distances between the transmitter and receiver coils, as indicated on the horizontal axis.

Figure 11a illustrates the Differential Non-Linearity (DNL) and the Integral Non-Linearity (INL) of the current DAC, when the measurement was made with a 2 kΩ resistor on the single-channel testing board. The highest differential non-linearity is under 0.3 Least Significant Bit (LSB), and the highest integral non-linearity is less than 0.6 LSB. Figure 11b shows the efficiency result of the stimulator with the charge pump included. The highest efficiency reaches 50%.

Figure 11c gives the output current versus the cross voltage between the different loads. The positive voltage will saturate to the power rail, and the negative voltage will finally saturate to a level determined by the charge pump, which is working at 1 MHz.

Figure 11d shows the wireless circuit measurement results. The longest working distance between the receiver coil and transmitter coil is 10 mm, while still stabilizing the load voltage at 3.3 V and delivering 25 mW to charge the battery.

**Figure 12:**
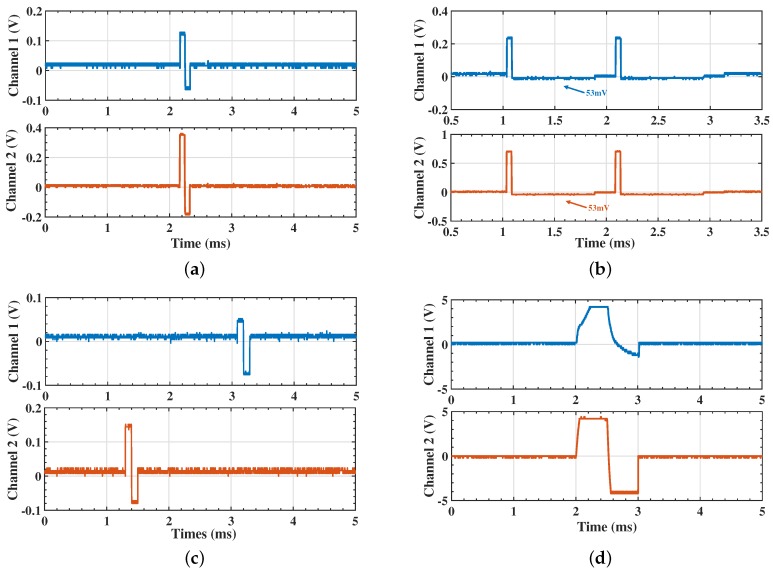
(**a**) The measured output voltage with a 5 kΩ load; (**b**) the measured independent output voltage under asymmetrical output mode from two different pairs of electrodes with different loads of 1 kΩ and 2 kΩ, respectively; (**c**) the measured output voltage; the stimulation current is programmed by the SPI; (**d**) in vitro experimental results. (Upper waveform) Output voltage over a pair of electrodes immersed in saline (lower waveform). Output voltage with tungsten electrode connected.

Finally, Figure 12 shows the dynamic result of the stimulation pattern. In Figure 12a, a 5 kΩ resistor is applied as the load with a symmetrical pattern. Figure 12b illustrates the output from site0 and site1 (Channel 1) and the output from site7 and site8 (Channel 2) with an asymmetrical pattern. In Figure 12c, a stimulator is programmed by the SPI to stimulate from site3 to site6 (Channel 1) at 5 kΩ and site13 to site10 at 2 kΩ. Figure 12d shows the captured stimulating pulse while the electrode is immersed in saline with the output connected to a tungsten electrode in the upper waveform. The bottom waveform illustrates the output from a different channel with a floating electrode for comparison purposes. In this picture, the voltage reaches the power rail after 0.5 ms, which is satisfactory for most applications. Table 2 summarizes the performance of the proposed work based on experimental results. A comparison to the state-of-the-art design is included, as well.

An in vivo experiment has been performed on the motor cortex of anesthetized mice. The mice were firstly anesthetized with pentobarbital (i.p. 80 mg/kg) and mounted in a stereotactic apparatus (RWD, 68001, Shenzhen, China). Erythromycin eye ointment was used to prevent eye drying, and an electric heating pad was applied to maintain the body temperature of mice around 37 Celsius. The skin over the midline was cut by sterile scissors and forceps to expose bregma, lambda, as well as the skull surface over the motor cortex. A small hole through the skull surface of the targeted area was drilled to load the tungsten electrode. Continuous stimulations with a 2-Hz frequency, a 40-ms duration and a 200-μA intensity were applied to the mice. The stimulation evoked reliable and time-locked vocalization and tail-swing, which disappeared right after the cessation of stimulation, strongly indicating the effectiveness of our system in vivo.

## 5. Conclusions

In this work, a wireless, implantable, multi-channel, programmable stimulator with arbitrary channel combination system has been designed. The size of the entire system is only 14 mm × 14 mm × 4 mm (without battery) including the IC fabricated in 0.18-μm TSMC HV CMOS technology. The proposed work enables wireless communication for the control of stimulation patterns and wireless battery charging. A novel channel management module using a switch array is presented, enabling arbitrary channel configuration with a reduction of 81% of switches as compared to a MUX-based channel selection. In vitro and in vivo measurement have been presented, as well.

## Figures and Tables

**Table 1:** Configuration of the on-chip registers for digital control. Pos: positive, Neg: negative, Int: the interval in Figure 4c, Clr: clear time.

Configure Information	Resolution (Bits)
Cycle Time	24
Numbers of Burst per Cycle	8
Positive Pulse Width	12
Positive Pulse Height	6
Negative Pulse Width	12
Negative Pulse Height	6
Time between Pos and Neg (Int)	8
Time between Two Burst (Clr)	8
Common Current Reference	2

**Table 2:** Comparison table.

Author	Jiang [33]	Van [34]	Jiang [35]	This work
Publication	15 TBioCAS	16 TBioCAS	16 ISCAS	-
CMOS Technology	0.6 μm	0.6 μm	0.6 μm	0.18 μm
Chip Area (mm${}^{2}$)	21.42, 3.9 *	2.1×1.6	-	1.5 × 2.5
System Area (mm${}^{3}$)	55 × 25 × -	-	46 × 46 × 8	14 × 14 × 3 **
Channel of Stimulator	3	8	3	8
Electrodes	24	16	24	16
Power Source	Inductive link	Battery	Inductive link	Inductive Link/Battery
HV Generation	rectifier	External	rectifier	Integrated CP
$I_{stim}$	≤1 mA	≤10 mA	≤3 mA	≤2 mA
Output Impedance	-	1 kΩ	-	9.1 MΩ
Working Distance of Inductive Link (mm)	-	-	11	10

* Master ASIC and two slave ASIC; ** the size is without the battery.
